# Conversation Electrified: ERP Correlates of Speech Act Recognition in Underspecified Utterances

**DOI:** 10.1371/journal.pone.0120068

**Published:** 2015-03-20

**Authors:** Rosa S. Gisladottir, Dorothee J. Chwilla, Stephen C. Levinson

**Affiliations:** 1 Language and Cognition Department, Max Planck Institute for Psycholinguistics, Nijmegen, Gelderland, The Netherlands; 2 Donders Institute for Brain, Cognition and Behaviour, Radboud University, Nijmegen, Gelderland, The Netherlands; The University of Nottingham, UNITED KINGDOM

## Abstract

The ability to recognize speech acts (verbal actions) in conversation is critical for everyday interaction. However, utterances are often underspecified for the speech act they perform, requiring listeners to rely on the context to recognize the action. The goal of this study was to investigate the time-course of auditory speech act recognition in action-underspecified utterances and explore how sequential context (the prior action) impacts this process. We hypothesized that speech acts are recognized early in the utterance to allow for quick transitions between turns in conversation. Event-related potentials (ERPs) were recorded while participants listened to spoken dialogues and performed an action categorization task. The dialogues contained target utterances that each of which could deliver three distinct speech acts depending on the prior turn. The targets were identical across conditions, but differed in the type of speech act performed and how it fit into the larger action sequence. The ERP results show an early effect of action type, reflected by frontal positivities as early as 200 ms after target utterance onset. This indicates that speech act recognition begins early in the turn when the utterance has only been partially processed. Providing further support for early speech act recognition, actions in highly constraining contexts did not elicit an ERP effect to the utterance-final word. We take this to show that listeners can recognize the action before the final word through predictions at the speech act level. However, additional processing based on the complete utterance is required in more complex actions, as reflected by a posterior negativity at the final word when the speech act is in a less constraining context and a new action sequence is initiated. These findings demonstrate that sentence comprehension in conversational contexts crucially involves recognition of verbal action which begins as soon as it can.

## Introduction

Wittgenstein invited us to imagine a world in which language serves no purpose; utterances are exchanged but lack functionality [1]. Such a community—if one could call it such—would lack *speech acts* [2–4]. Just like other aspects of social behaviour, conversation involves verbal actions such as requests, greetings and complaints [5]. The exchange of such speech acts in everyday conversation is the core ecology for language—this is where children acquire language and the great bulk of language usage occurs.

The prime task in conversation is to recognize what speech act is being performed and prepare a fitted reply. *Speech act recognition*, the topic of this study, is the process of recognizing the action of an utterance in a given context. In some cases speech act recognition may be quite direct, particularly in ritualized expressions where there is a one-to-one correspondence between the form of the utterance and its speech act function (like *Gesundheit*! in response to a sneeze). Syntactic features such as wh-question words (*who*, *when*), imperatives (*put*, *go*) and interrogative word order can likewise provide clues about the relevant speech act. Since the action level of meaning is sometimes referred to as *illocutionary force* [2], such speech act clues have been called *illocutionary force indicators* [3,4,6]. However, listeners can’t always rely on such morphosyntactic clues. Interrogatives and imperatives, for instance, can perform many other actions than questioning and requests [7]. Declarative statements offer even less of an indication, being sometimes radically underspecified for the action. For example, a declarative utterance like *I have a car* could be used to offer somebody help with moving, to indirectly reject an offer for a ride, or to answer a question about commuting. The utterance is thus compatible with multiple speech acts and listeners have to rely on the context to recognize which action is being performed. This problem of *underspecification at the action level* is pervasive in everyday conversation.

The challenge for participants in regards to speech act recognition is further enhanced by the very tight time constraints in turn-taking. The most frequent gaps between turns in conversation are only around 200 ms [8–10]. A gap of 200 ms does not leave much time for both recognizing the action in the prior turn and planning a response to it. In fact, findings from word production experiments indicate that it takes people at least 600 ms just to plan a one-word utterance [11] and even longer for sentences with multiple words [12]. These timing facts imply that listeners start planning their responses before the prior speaker has finished speaking, and since the appropriateness of the response crucially depends on recognizing the speech act, they suggest that speech act recognition must be an early process [3]. On this *early speech act recognition account*, recognition of the action is not made at the final stage in the comprehension process, occurring at the last word of incoming utterances, but takes place early on when the turn has only been partially processed. The primary aim of the present study was to test the early speech act recognition account, investigating the time-course of verbal action understanding.

Given that the turn construction doesn’t always give us indications about what speech act is coming up, how could early recognition of the action be made? One source of information that can constrain the speech act possibility space is the *sequential context*, i.e., preceding turns in the conversation. Speech acts do not exist in a vacuum; they are coherently organized into larger action sequences [5]. One of the basic action sequences is the *adjacency pair* [5,13], where the first utterance puts powerful constraints on the following turn. The first part in an adjacency pair “projects a prospective relevance, and not only a retrospective understanding. It makes relevant a limited set of possible second pair parts, and thereby sets some of the terms by which a next turn will be understood” [5]. Questions, for instance, are followed by answers, and invitations call for acceptances or rejections. Prior talk in conversation is thus not merely the background to speech act comprehension, but rather contains a rich structure of action sequences that could proactively funnel possible interpretations of upcoming talk. If this notion is correct, participants in conversation can profit from implicit knowledge of action sequences during speech act comprehension.

The goal of the present experiment was twofold: a) to investigate the time-course of speech act recognition in utterances that are underspecified for the action, and b) examine the effects of sequential context on speech act recognition. The larger theoretical relevance of this investigation is that it addresses the fundamental problem of how listeners map speech act functions onto underspecified utterances—a core cognitive ability that gives language its basic functionality.

### Prior research

Several strands of research have addressed speech act comprehension broadly construed. Eye-tracking experiments have, for instance, demonstrated early sensitivity to speech act function, even in young children [14–18]. However, these studies are not informative about the key focus of the present experiment, namely how underspecified utterances (without any morphosyntactic speech act clues) are rapidly understood as performing certain actions. Another strand of research has focused on *indirect speech acts* [19], that is speech acts that clearly require substantial inferencing to be understood. FMRI studies on indirect replies [20] and indirect requests [21] report activations not only in typical language regions but also areas implicated in mentalizing and affective empathy, as well as action planning and motor control. These studies provide important insights into the neural substrates of indirect speech act comprehension, but due to the poor temporal resolution of fMRI cannot tell us much about the time-course of speech act recognition.

A growing body of research has made use of the excellent temporal resolution of EEG and MEG to investigate pragmatic language comprehension in real time. Studies on non-conversational discourse have shown that spoken or written words are related to the wider discourse context extremely rapidly, from 150 ms after word onset [22,23]. However, research on pragmatic inferencing—which may play a role in speech act recognition—indicates that language processing at the level of pragmatics is not always so fast. For instance, understanding irony involves late inferential processes, reflected by modulations of the P600 component [24,25], and scalar inferences are associated with N400 effects only in some cases [26,27]. The above studies highlight that pragmatic language comprehension involves both early and late processes. Importantly, they do not address comprehension at the speech act level, i.e., the processing of utterances in conversational contexts, so their relevance for the time-course of speech act recognition is unclear.

Turning to research that comes closer to dialogue, a recent EEG study investigated speech act processing in written words that performed either a requesting or a naming speech act depending on a prior video-taped context sentence (e.g., What are these called?/What can I get you?—*Plant*) [28]. The brain responses for the two speech acts diverged as early as 120 ms after the onset of the critical words. A follow-up MEG study [29] reported that the requests engaged comprehension systems in the right hemisphere within 100 ms after word onset, followed by theory of mind activations in the medial prefrontal and temporo-parietal areas from 200 to 300 ms. Naming speech acts, on the other hand, activated brain areas involved in lexico-semantic retrieval from 100 to 150 ms. In a study on visually presented (i.e., written) indirect requests (e.g., *My soup is too cold to eat* in a restaurant context), ERP differences between indirect requests and literal statements were found from the second word onwards, but no differences were present at the final word [30]. These EEG and MEG findings provide some supportive evidence for the early speech act recognition account, at least in the visual modality. However, these studies are far removed from conversation and overlook the importance of spoken language input. When a sentence is visually presented, one word at a time, the linguistic signal is artificially spread out over a much longer time period than in spoken language (or natural reading for that matter). This is problematic for investigating the time-course of speech act recognition, as effects of sentence-level factors such as speech act function may not be confined to a single word. Although a few studies have indirectly addressed spoken speech act comprehension by investigating processing of prosody in question-answer dialogues [31–33], to our knowledge there are no studies directly investigating the time-course of speech act processing using auditory stimuli. A critical next step therefore is to shed light on speech act recognition in spoken language, the prime modality of natural conversation.

### The present study

Against this background, several important questions need to be addressed. What is the time-course of speech act recognition in the auditory modality? In light of the extraordinarily fast transitions between turns in conversation, how quickly can listeners recognize the speech act in action-underspecified utterances? How does the type of action and how it fits into the action sequence influence this process? We used ERPs to investigate these issues, using spoken dialogues approximating informal everyday conversation.

The experimental paradigm was designed based on the following criteria. First, in order to examine how listeners recognize speech acts in utterances that are underspecified for the action, the critical utterances do not contain morphosyntactic speech act clues such as question words or imperative verbs. Secondly, to get a better understanding of the role of sequential context, we used speech acts that differ in how they fit into the larger action sequence (for details, see below). Third, since passively overhearing a dialogue is quite different from taking an active part in one [34], we use an action categorization task to mimic the response demands and attention level necessary for everyday interaction. While the absence of a behavioural task is appropriate for ERP studies on passive reading or listening to non-conversational discourse, the crucial task in conversation is comprehending-for-responding. As we have argued above, a critical part of that task is identifying the speech act category of the incoming turn (see also [3]).

Examples of stimuli in Dutch and English translations are presented in Table 1. The dialogues contain target utterances (e.g., *I have a credit-card*) that deliver three functionally distinct speech acts (Answer, Pre-offer, Declination) depending on the prior turn. The Answer condition involves a question-answer sequence (*How are you going to pay for the ticket?—I HAVE A CREDIT-CARD*). The Answers serve as the control condition as they should be easiest to comprehend. This assumption is supported by the results of a self-paced reading study discussed below [35], in which reading times were shortest for Answers on all measures. The Declination condition consists of an offer, followed by a rejection (*I can lend you money for the ticket.—I HAVE A CREDIT-CARD*). The Pre-offer condition contains a first turn expressing need or desire for something, followed by a prelude to an offer, called a *pre-offer* [5,36] in conversation analysis (*I don’t have any money to pay for the ticket.—I HAVE A CREDIT-CARD*). To balance control and variety in the format of the critical utterances, we divided the dialogues in two stimulus sets; Set 1 contains utterances starting with *I have*… but Set 2 includes other verbs (see Table 1 and Methods). In none of the conditions can listeners rely on clues in the utterance to recognize the speech act. Instead, it is the sequential context, that is the prior turn, which determines the action. Since the target sentences are identical across conditions, ERP differences between them can be attributed to their speech act function and how listeners arrive at that function. This design allows an investigation of speech act recognition in spoken dialogues that do not contain semantic or pragmatic anomalies.

**Table 1:** Stimuli in Dutch and English translations.

	Set 1		Set 2	
Condition	Context	Target Utterance	Context	Target Utterance
Answer **(Control)**	Hoe ga je voor het ticket betalen?	Ik heb een creditcard.	Waar koop je je shampoo?	Ik ga naar de Kruidvat.
	*How are you going to pay for the ticket*?	*I have a credit-card*.	*Where do you buy your shampoo?*	*I go/am going to the Kruidvat [drugstore].*
Declination **(Context highly constraining + target utterance ends the sequence)**	Ik kan je wat geld lenen voor het ticket.	Ik heb een creditcard.	Ik kan wel shampoo voor je meenemen?	Ik ga naar de Kruidvat.
	*I can lend you money for the ticket*.	*I have a credit-card*.	*I can bring some shampoo for you?*	*I go/am going to the Kruidvat [drugstore].*
Pre-offer **(Context less constraining + target utterance starts a new sequence)**	Ik heb geen geld om het ticket te betalen.	Ik heb een creditcard.	Mijn shampoo is op.	Ik ga naar de Kruidvat.
	*I don’t have any money to pay for the ticket*.	*I have a credit-card*.	*My shampoo is finished.*	*I go/am going to the Kruidvat [drugstore].*

An important aspect of the design is that while the critical actions—Declinations and Pre-offers—are both relatively indirect, they differ in how they fit into the larger action sequence. Declinations are second parts of adjacency pairs (see above), which entails that the context turn (first part of the pair) should be highly constraining in terms of what type of action can follow. An offer, for instance, sets up a normative expectation for an acceptance or declination. Out of the vast possibility space for speech acts in conversation, the prior turn has narrowed the likely actions down to two. This is not the case for Pre-offers. Although typically responding to a telling of some trouble, pre-offers do not close an adjacency pair but rather initiate a new sequence, a so-called *pre-sequence* [5,36]. Pre-sequences are preliminary to the main course of action, in this case a more direct offer if the conversation were to continue. This is illustrated below (for a similar example from a real conversation, see [5]):

1 A:         I don’t have any money to pay for the ticket.
2 B:  *Pre-Offer*      I have a credit card.
3 A:  *Go-ahead*     You do?
4 B:  *Offer*       Want to use it?
7 A:  *Acceptance*   Yeah.

Understanding Pre-offers may therefore involve forward inferences about upcoming talk, akin to causal consequence inferences in text processing [37]. An additional difference between Pre-offers and Declinations is that the context turn is less constraining in Pre-offers. The first turn in the Pre-offer dialogues can be followed by a large number of actions; there is no normative expectation for a Pre-offer. The utterance *I don’t have any money* could, for instance, be followed by responses such as condolences (*Oh dear*, *That sucks*), a telling of one’s own experience (*Me neither*), or a suggestion (*Why don’t you ask somebody for a loan*?); a direct offer or a pre-offer are just open possibilities. Thus by comparing Pre-offers and Declinations we can go beyond the traditional distinction between direct and indirect speech acts and investigate how speech act recognition is modulated by the type of action being performed and how it fits into the sequential context (high- vs. low-constraining context, end of an action sequence vs. a start of a new one).

We have previously reported a behavioural study using the same dialogues, in which the target replies were presented visually in self-paced reading [35]. After each dialogue, participants were asked to categorize the action in the target sentences (*I have a credit-card*) as doing answering, offering or declining. The categorization accuracy was very high (95.8%), indicating that listeners are very good at identifying the speech act in such action-underspecified sentences. Reading times were longer in Pre-offers than Answers at the first word, while Declinations took longer at the verb and the final word, relative to Answers (unpublished data). However, the differences in reading times were very small and standard deviation large, preventing conclusive interpretations. The ERP method has advantages over self-paced reading in that it is compatible with spoken language input and can indicate with much higher precision the time-course of differences between experimental conditions. Moreover, ERPs allow to determine whether there are quantitative or qualitative processing differences between the actions [38]. Given that Declinations and Pre-offers have different properties, it is possible that they recruit in part qualitatively different comprehension processes. If so, this should be reflected by differences in the scalp distribution of the ERP effects to Declinations vs. Pre-offers.

If the early speech act recognition account is correct and auditory speech act recognition takes place early in the turn when the utterance has only been partially processed, then the three speech acts should differ early on in the target utterance (*I have a credit-card*), for instance at the first word (*I*) or the verb (*have*). Moreover, there should be no ERP differences at the utterance-final word, i.e., *credit-card* (note that this is one word in Dutch, the language of the stimuli); if the action has already been recognized at that point, the processing of the final word should only add to the propositional meaning of the utterance, which for each target is the same for all three conditions and hence no differences should occur at the final word. Our predictions for the experiment were therefore as follows. We expected that both Declinations and Pre-offers, the critical conditions, would elicit ERP effects relative to the control condition (Answers) in an early time-window corresponding to the first word and the verb, and not at the final word. The critical questions are how early the effects appear and whether there are quantitative or qualitative ERP differences between Pre-offers and Declinations, reflecting that the type of action and sequential context influences speech act recognition. Given that Pre-offers have a more complex action sequence structure (the context is less constraining and the target utterance initiates a new sequence), we hypothesized that they might elicit additional ERP effects relative to Declinations. We did not have specific predictions regarding the ERP components, given that the two visual ERP experiments on speech act recognition discussed above [28,30] do not yield a clear picture in terms of which ERP components are involved. However, we speculated that frontal ERP effects would be involved, as both studies [28,30] report frontal effects.

## Methods

### Participants

Forty-four right-handed speakers of Dutch with no hearing or speech problems and normal or corrected-to-normal vision were recruited from the subject database of the Max Planck Institute for Psycholinguistics in Nijmegen (28 female, 16 male, mean age 20, age range 18–27). The study was approved by the Ethische Commissie Gedragswetenschappelijk Onderzoek at Radboud University Nijmegen. Participants gave written informed consent according to the Declaration of Helsinki prior to the study and were paid 8 Euro per hour for their participation. EEG data from two participants were removed from analysis due to excessive artifacts.

### Construction of materials

The stimuli are auditory versions of the dialogues described in Gisladottir et al. [35]. We created 378 two-utterance long, naturalistic dialogues in Dutch reflecting informal daily conversations between friends or relatives. The discourse topics include buying groceries, going out, and working/studying. Each dialogue consists of a critical utterance and a preceding context utterance, which biases the interpretation of the critical utterance as an Answer, Declination or Pre-Offer (see examples in Table 1). In total there are 126 critical utterances, presented in three contexts (conditions). The number of words in the utterances ranges from 3 to 7 words (median: 4 words), and average utterance duration is 1175 ms. The targets are constructed such that the final word is critical for understanding the propositional meaning of the utterance, irrespective of speech act function. In order to balance control and variety in the stimulus materials, half of the target sentences start with “I have” (Dutch *ik heb*), e.g., “I have a credit-card” (Set 1). The other half was more varied and included utterances like “I am going to the market” and “My brother is a mechanic” (Set 2). To maintain consistency in the way the Declinations and Pre-offers were connected to their contexts, we ensured that there was at least one clear implicated premise and an implicated conclusion for each sentence-pair [39]. According to theories of linguistic pragmatics, when presented with an utterance that is indirect, the hearer needs to access an implicated premise and combine it with the proposition expressed to derive the implicated conclusion [39]. In the dialogue (A): *I can lend you money for the ticket*.–(B): *I have a credit-card*, the implicated premise is that a credit-card can pay for things, including tickets. The implicated conclusion is that speaker B does not need A’s help with paying for the ticket.

The sentences were recorded in a soundproof room at 44.1 kHz sampling rate and 16-bit resolution. Four native speakers of Dutch (two male, two female) were instructed to act out the written dialogues as naturally and informally as possible in four different pairings (male1-male2; female1-female2; male1-female1; male2, female2). The partners of each pair took turns in acting context utterances and critical utterances. The context utterances were extracted from those recordings, while the critical utterances were recorded separately from a list (without context) to prevent the prosody of the critical utterance from biasing one condition over another. The overall sound intensity of the recordings was normalized to prevent loudness differences between the items. The stimuli were pseudo-randomized and balanced across three lists, such that participants heard each critical utterance only once. Each list contained 126 dialogues with an equal number of trials across conditions and stimulus sets. Care was taken that the voices of the native speakers appeared as equally as possible in each action within each list.

### Procedure

Participants were given written instructions that included one example of each action. They were instructed to pay attention to the underlying meaning of the responses in the dialogues (the target utterances) and answer a comprehension probe: after each dialogue, participants indicated with a mouse click what the second speaker was doing with his response. For this task the options were Answering, Offering and Declining (Dutch *antwoorden*, *aanbieden*, *weigeren*). Since pre-offer is not a colloquial term, the broader concept of offering was chosen. Participants were seated in a comfortable chair in a soundproof room facing a computer monitor. There were 126 experimental trials presented auditorily through loudspeakers, preceded by 18 practice items. On each trial a fixation cross appeared in the middle of the screen which lasted throughout the entire dialogue. Participants were instructed to avoid eye movements and other movements during the presentation of the fixation cross. The context utterance was played 500 ms after the appearance of the fixation cross, followed by a 250 ms pause before the target recording was played. In order to prevent an abrupt start and ending of the sentences, the recordings included a 50 ms buffer before sentence onset and after offset, such that the pause between context and target was in total 350 ms. This pause is similar to average gap durations reported in corpus studies of Dutch, which range from 8 to 380 ms depending on the study [9,10,40]. The fixation cross disappeared 1200 ms after the offset of the target utterance recording. A blank screen was then presented for 1500 ms, until the comprehension probe was presented. This delayed task reduced contamination of the ERPs of interest by movement related EEG activity. Upon answering the comprehension probe (see above) a blank screen appeared for 2000 ms and then the next trial began. The trials were presented in 6 blocks, allowing participants to make eye movements and rest between them. After the EEG recording, participants filled out the Empathy Quotient questionnaire (EQ) [41] (see S2 File in Supplementary Information).

### Electrophysiological recordings

The EEG was recorded with 36 active electrodes mounted in a cap (actiCap), referenced to the left mastoid. After the recording, the data were re-referenced off-line to the average of the left and right mastoids. Vertical eye movements were monitored with an electrode placed below the left eye and an electrode in the cap right above the left eye (Fp1). Horizontal eye movements were monitored through two electrodes in the cap placed approximately at the left and right outer canthi (F9 and F10). Bipolar EOGV and EOGH was computed. Electrode impedances were kept below 20 KΩ. EEG and EOG data were amplified with a bandpass filter of. 02 to 250 Hz with a 10 second time constant and digitized at a sampling frequency of 500 Hz. Recording and analyses were performed with Brain Vision Analyzer.

## Results

### Behavioural analysis and results

Behavioural responses from all participants included in the EEG analysis were analyzed (42 out of 44 participants). Mean accuracy in the action categorization task was very high, 96.6% (SD 5.4%) (see Table 2). Participants correctly categorized 98.5% of Answers, 94.8% of Pre-offers and 96.5% of Declinations. The accuracy data were analysed with mixed-effects logistic regression using the lme4 package [42] in the statistics software R [43]. Mixed-effects logistic regression is better suited for the analysis of categorical outcome variables (such as question-answer accuracy) than ANOVA and allows the inclusion of participants and items as random factors in a single analysis [44]. The fixed effects were Action and Set; while the Set factor (see Construction of materials) was not meant to test any hypothesis of theoretical relevance, it was included in the analysis to check for interactions between action and the linguistic form of the sentence. We used the most maximal random effects structure justified by the experimental design and for which convergence was reached [45]. This included random intercepts by participant and item, as well as by-participant random slopes for Action and Set and by-item random slopes for Action. For model comparison, see supplementary information (S1 File). The full model with Action, Set and the Action by Set interaction indicated that Declinations were categorized less accurately than Answers (Estimate: -1.57, SE: 0.40, *z* = -3.94, *p* <. 001) and Pre-offers were categorized less accurately than Answers (Estimate: -1.48, SE: 0.43, *z* = -3.47, *p* <. 001). However, the comparison between Declinations and Pre-offers was not significant (Estimate: -0.26, SE: 0.27, *z* = -0.94, *p* = .34). An Action by Set interaction reflected that Pre-offers in Set 1 were categorized more accurately than in Set 2 (Estimate: 0.84, SE: 0.38, *z* = 2.22, *p* <. 05).

**Table 2:** Behavioural results.

	Answer	Declination	Pre-offer
Overall accuracy	98.5% (2%)	96.5% (3.8%)	94.8% (8%)
Set 1 accuracy	98.0% (3.7%)	96.2% (4.5%)	97.2% (5.2%)
Set 2 accuracy	99.1% (2.2%)	96.9% (4.9%)	92.4% (11.9%)

Mean accuracy (and standard deviation) in the categorization task for all items (overall) and for each stimulus Set.

### ERP analysis

The EEG data were averaged relative to the first word onset and the sentence-final word onset of the target utterance. For each time-locking point the EEG data were segmented into epochs of 1200 ms with a 150 ms pre-stimulus baseline. Artifacts were removed by excluding epochs with excessive EEG (>100 μV) or EOG (>75 μV) amplitude. In the final dataset of 42 participants, 17% of trials were rejected due to artifacts. The percentages of rejected trials did not differ across the three experimental conditions at the two time-locking points (*Fs* < 1.5, *p*s >. 23). Only artifact-free trials were included in the averages.

**Fig 1:**
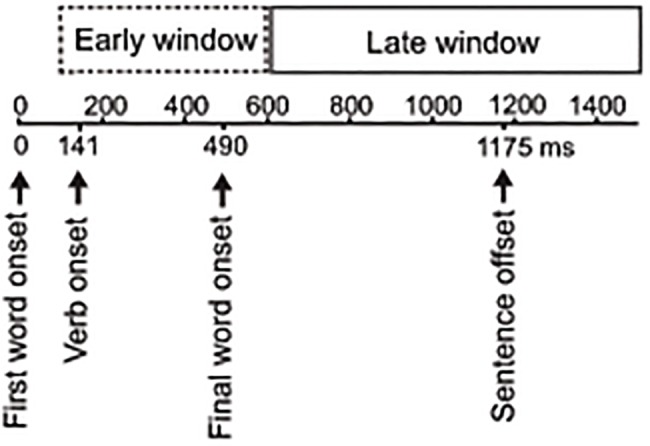
Timeline. Mean onset of the first word, verb and final word in target utterances and a rough timeline for the early and late utterance time-windows.

For a separation of ERP effects elicited early in the target sentences and effects elicited at the final word, we defined two broad time-windows: an *early utterance time-window* from 100 to 600 ms after first word onset and a *late utterance time-window* from 100 to 1000 ms after final word onset. These time-windows were chosen for the following reasons. The early utterance time-window covers the duration of the first word and the verb, and therefore captures early speech act recognition effects (the target sentences involve connected speech with subjects and verbs of relatively short duration; see Fig. 1). The final word of the target utterances occurs on average 490 ms after utterance onset; however, since the first 100 ms after word onset reflect exogenous, stimulus-bound ERP components, which are largely insensitive to cognitive factors [46,47], the first 100 ms of the final word should be very similar across experimental conditions. As a consequence, the endpoint of the early utterance time-window was set at 600 ms (roughly 490 ms + 100 ms), and the starting point of both early and late utterance time-windows was set at 100 ms after first and final word onset. Importantly, these two time-windows are not overlapping, as illustrated in Fig. 1.

**Fig 2:**
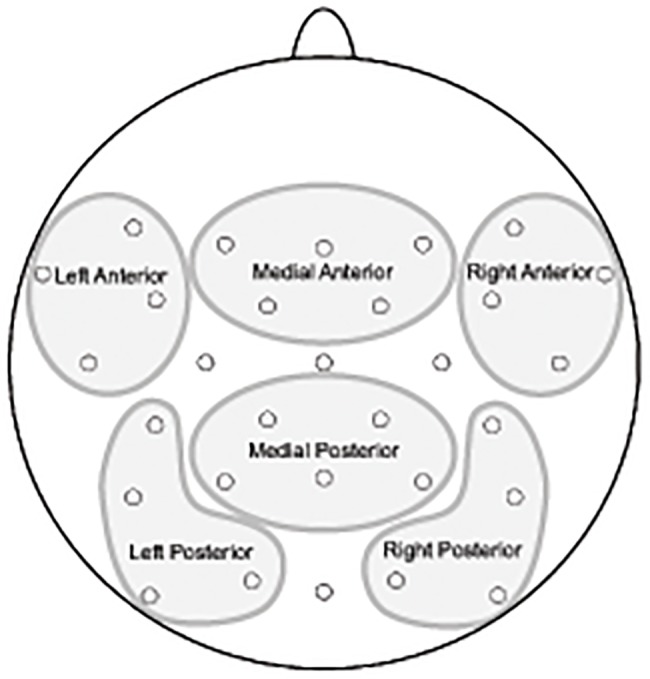
Regions used for analyses of EEG data.

To further narrow down relevant time-windows, we first performed omnibus repeated measures ANOVAs on mean amplitudes in 100 ms consecutive windows in the early and late utterance time-windows described above, using all electrodes; see S1 Table and S2 Table in supplementary information (for a similar approach, where consecutive time-windows of 100 ms were used to get a more fine-grained picture of the time-course of language-relevant ERP effects, see for example [48,49]). Based on these initial omnibus ANOVAs, follow-up analyses were performed in 100 ms consecutive windows at the first word from 100 to 600 ms, and at the final word from 100 to 200 ms and 600 to 1000 ms. The lateral ANOVA included the topographical factors AntPost (for Anterior vs. Posterior sites), Hemisphere and Site (i.e., electrode), yielding four regions of four electrodes each (for an illustration of the regions, see Fig. 2). The medial ANOVA included the topographical factors AntPost and Site, yielding two regions of five sites each. Note that the medial ANOVA included 8 lateral electrodes and only 2 midline electrodes, and is therefore referred to as medial. Since no Action×Set interactions were obtained in the initial omnibus ANOVA (see S1 Table and S2 Table in supporting information), Set was removed as a factor in these regional analyses. When applicable, the Greenhouse-Geisser correction was applied to correct for violations of the assumption of sphericity; original degrees of freedom are reported. Main effects of Action and relevant interactions in the regional omnibus analysis were followed up with pairwise comparisons (ANOVA), contrasting every action with each one of the others.

### ERP results

#### Early utterance time-window (first word onset of the target utterance)

**Fig 3:**
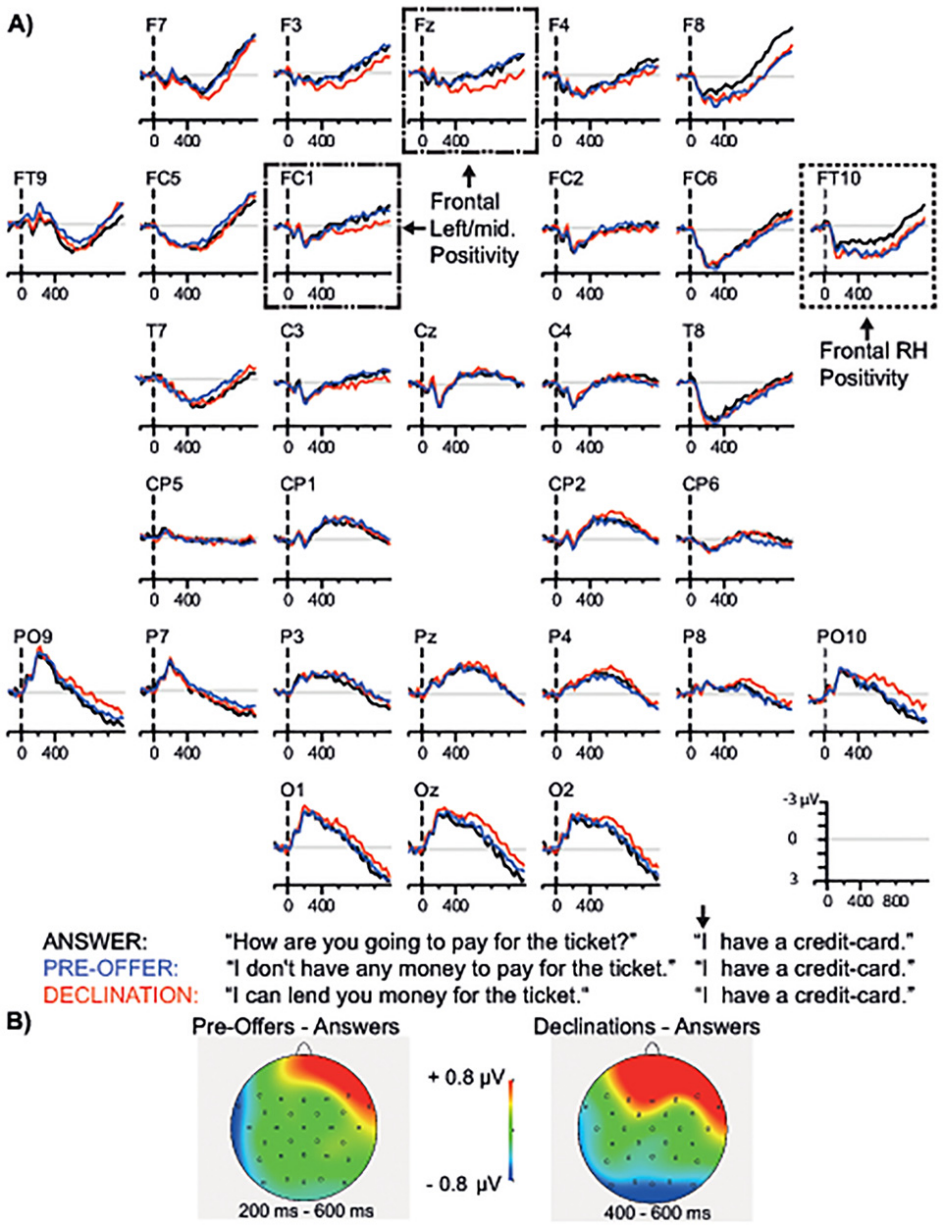
Early utterance time-window. A) Grand-averaged ERPs time-locked to the onset of the first word. Representative electrodes showing the relevant effects are highlighted in dashed boxes. B) Scalp distribution of the ERP effects in the early utterance time-window. All waveforms were low-pass filtered (10 Hz) for illustration purposes only. Negativity is plotted upwards.

Fig. 3 shows the grand averaged ERPs time-locked to the first word. All conditions elicited the early ERP components characteristic of auditory stimuli, the N1 and P2. The regional omnibus analysis at lateral electrodes (see Table 3) yielded Action×AntPost×Hemisphere interactions from 100 to 600 ms (all *F*s ≥ 3.69, *p*s <. 05) and Action×Hemisphere×Site interactions from 100 to 400 ms (all *F*s ≥ 2.55, *p*s <. 05). The medial omnibus ANOVA revealed an Action×AntPost interaction from 500 to 600 ms (*F*(2, 82) = 4.11, *p* <. 05). Pairwise comparisons between the actions were performed on the basis of these interactions.

**Table 3:** Regional omnibus analyses and pairwise comparisons for the early utterance time-window.

		Analysis	Source	DF	100–200	200–300	300–400	400–500	500–600
REGIONAL OMNIBUS		Lat	Action×AntPost×Hem	2, 82	4.14*	7.08**	3.69*	4.08*	5.64**
			Action×Hem×Site	6, 246	3.42*	2.81*	2.55*		
		Med	Action×AntPost	2, 82					4.11*
PAIRWISE COMPARISONS	D vs. A	Lat	Action×Hem ×Site	3, 123	3.88*	3.63*	3.94*		
			Action×AntPost×Hem	1, 41				6.77*	7.08*
			Action×AntPost×Site	3, 123				2.89*	
		Lat Ant	Action	1, 41					6.70*
			Action×Hem	1, 41				5.40*	4.59*
			Action×Hem×Site	3, 123				3.28*	3.06*
		Right Ant	Action	1, 41				8.29**	
		Med	Action×AntPost	1, 41					6.86*
		Med Ant	Action	1, 41					6.90*
	P vs. A	Lat	Action×Hem	1, 41				6.47*	7.99**
			Action×AntPost×Hem	1, 41	9.63**	15.18**	7.10*	5.46*	10.37**
			Action×AntPost×Hem×Site	3, 123	2.96*			3.40*	2.88*
			Action×Hem×Site	3, 123	7.68**	4.02*	3.21*		
		Lat Ant	Action×Hem	1, 41	4.77*	10.40**		8.56**	11.57**
			Action×Hem×Site	3, 123	6.93**	4.84**	4.08*	3.55*	
		Right Ant	Action	1, 41		7.94**	6.08*	10.61**	6.66*
	P vs. D	Lat	Action×AntPost×Hem	1, 41		5.18*			
		Med	Action×AntPost	1, 41					6.07*

D = Declination, A = Answer, P = Pre-offer, Lat = lateral regions, Med = medial regions, Ant = anterior regions, Hem = hemisphere, AntPost = Anteriority.

*p <. 05

**p<.01

#### Declination versus Answer

The lateral analysis (see Table 3) revealed that mean amplitude was more positive-going in Declinations than in Answers at the anterior region in the right hemisphere from 400 to 500 ms (Action; *F*(1,41) = 8.29, *p* <. 01), and at bilateral anterior sites from 500 to 600 ms (Action; *F*(1,41) = 6.70, *p* <. 05). Mean amplitude was also more positive-going in Declinations than in Answers at the anterior medial region from 500 to 600 ms (Action; *F*(1,41) = 6.90, *p* <. 05).

#### Pre-offer versus Answer

The lateral analysis revealed that mean amplitude in Pre-offers was more positive-going than in Answers at the anterior region in the right hemisphere from 200 to 600 ms (Action; *F*s ≥ 6.08, *p*s <. 05). The medial ANOVA did not yield any reliable differences across conditions (*F*s ≤ 2.34, *p* >. 06).

#### Pre-offer versus Declination

The lateral ANOVA revealed an Action×AntPost×Hemisphere interaction from 200 to 300 ms (*F*(1,41) = 5.18, *p* <. 05), but no reliable differences were obtained in follow-up analyses (*F*s ≤ 2.35, *p*s >. 08). In the medial analysis, mean amplitude tended to be more positive-going in Declinations than in Pre-offers at the anterior medial region from 500 to 600 ms (Action; *F*(1,41) = 3.75, *p* = .06).

#### Summary of ERP effects in the early utterance time-window

As predicted, there were early ERP differences between the actions in the early utterance time-window (corresponding to the first word and the verb). In particular, Declinations and Pre-offers both showed a frontal positivity, relative to Answers. In Pre-offers the positivity was restricted to the right hemisphere from 200 to 600 ms. In Declinations the positivity was present at anterior sites in the right hemisphere from 400 to 500 ms and extended to anterior bilateral and medial regions from 500 to 600 ms. The frontal positivity in Declinations was marginally significant relative to Pre-offers at the anterior medial region from 500 to 600 ms (*p* = .06). Differences in scalp distribution of the frontal positivity in Declinations and Pre-offers are illustrated in the topographical maps in Fig. 3.

### Late utterance time-window (final word onset of the target utterance)

**Fig 4:**
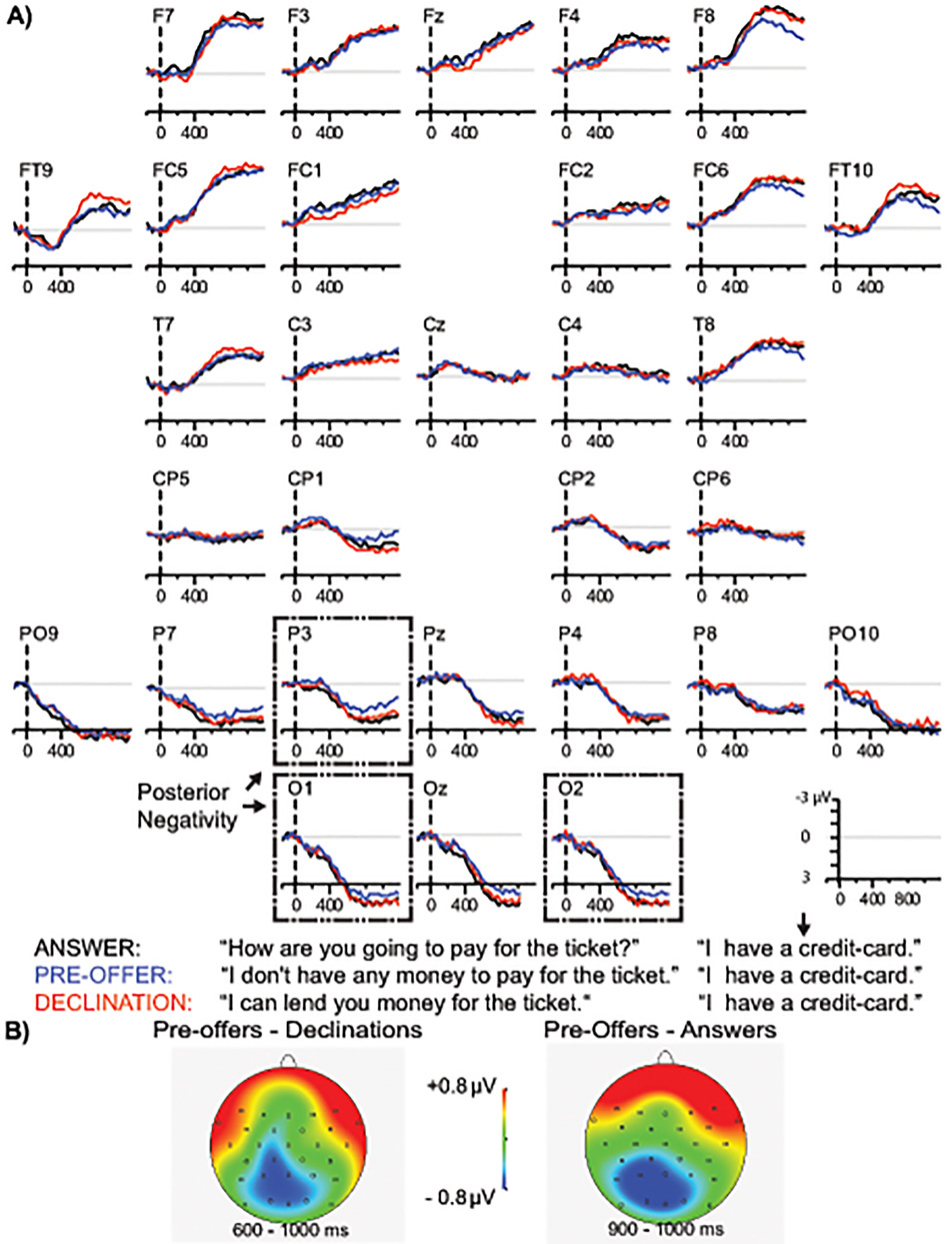
Late utterance time-window. A) Grand-averaged ERPs time-locked to the onset of the final word. Representative electrodes showing the relevant effects are highlighted in dashed boxes. B) Scalp distribution of the ERP effects in the late utterance time-window.

Fig. 4 presents the waveforms aligned to the final word onset. The regional omnibus analysis for medial sites (see Table 4) yielded an Action×Site interaction from 100 to 200 ms and 600 to 700 ms (*F*s ≥ 2.56, *p*s <. 05) and an Action×AntPost interaction from 900 to 1000 ms (*F*(2,82) = 3.57, *p* <. 05). In the lateral analysis an Action×AntPost interaction was obtained from 600 to 1000 ms (*F*s ≥ 3.43, *p*s <. 05) in addition to an Action×Site interaction from 700 to 800 ms (*F(*6,246) = 2.65, *p* <. 05). Hence follow-up comparisons between the actions were performed at medial sites from 100 to 200 ms, 600 to 700 ms and 900 to 1000 ms and at lateral sites from 600 to 1000 ms.

**Table 4:** Regional omnibus analyses and pairwise comparisons for the late utterance time-window, for those epochs that showed significant effects.

		Analysis	Source	DF	100–200	600–700	700–800	800–900	900–1000
REGIONAL OMNIBUS		Lat	Action×AntPost	2, 82		3.78*	3.43*	4.17*	4.21*
			Action×Site	6, 246			2.65*		
		Med	Action×Site	8, 328	3.47**	2.56*			
			Action×AntPost	2, 82					3.57*
PAIRWISE COMPARISONS	D vs. A	Med	Action×AntPost	1, 41	6.32*				
	P vs. A	Lat	Action×AntPost	1, 41			4.41*	6.79*	8.75**
		Lat Post	Action	1, 41					5.11*
		Med	Action×AntPost	1, 41					5.56*
		Med Post	Action	1, 41					4.97*
	P vs. D	Lat	Action×AntPost	1, 41		7.64**	6.45*	9.04**	8.52**
		Lat Post	Action	1, 41		9.26**	4.24*	4.40*	5.93*
		Med	Action×AntPost	1, 41					4.28*
		Med Post	Action	1, 41					6.31*

D = Declination, A = Answer, P = Pre-offer, Lat = lateral regions, Med = medial regions, Post = posterior regions, AntPost = Anteriority.

*p <. 05

**p<.01

**Declination versus Answer**. The medial ANOVA (see Table 4) yielded an Action×AntPost interaction from 100 to 200 ms (*F*(1,41) = 6.32, *p* <. 05), but follow up analyses did not reveal significant differences between the actions (*p*s >. 12). In the lateral ANOVA there were no significant differences (*Fs* ≤ 2.34, *p*s >. 10).

**Pre-offer versus Answer**. The lateral analysis revealed that mean amplitude was more negative-going in Pre-offers than in Answers at bilateral posterior regions from 900 to 1000 ms (Action; *F*(1, 41) = 5.11, *p* <. 05). In the analysis for medial sites, mean amplitude was also more negative-going in Pre-offers than in Answers in the medial posterior region from 900 to 1000 ms (Action; *F*(1, 41) = 4.97, *p* <. 05).

**Pre-offer versus Declination**. Mean amplitude was more negative-going in Pre-offers than in Declinations at bilateral posterior regions from 600 to 1000 ms (Action; *F*s ≥ 4.24, *p* <. 05). Mean amplitude was also more negative-going in Pre-offers than in Declinations at medial posterior sites from 900 to 1000 ms (Action; *F*(1, 41) = 6.31, *p* <. 05).

**Summary of ERP effects in the late utterance time-window**. As expected, there were no differences between Declinations and Answers in the late utterance time-window. Pre-offers, however, elicited a late, posterior negativity relative to both Answers and Declinations. Relative to Declinations, the negativity was present at posterior bilateral regions (600 to 1000 ms) and the posterior medial region (900 to 1000 ms). Relative to Answers, the negativity was present at posterior bilateral and medial regions from 900 to 1000 ms. The scalp distribution of these effects is illustrated in the topographical maps in Fig. 4.

## Discussion

The aim of this article was to address a fundamental problem in conversation, namely how utterances that are underspecified at the action level can quickly be understood as performing certain speech acts. This study goes beyond prior research by investigating the time-course of speech act recognition in such action-underspecified utterances in spoken dialogues and exploring the effect of sequential context on this process. The target utterances were identical across conditions but differed in the type of speech act performed and how it fit into the larger action sequence (high- vs. low-constraining context, end of an action sequence vs. a start of a new one). The behavioural results indicate that even for action-underspecified utterances participants are very good at identifying the underlying speech act; the mean accuracy in the comprehension task was 96.6%. The ERP results show that speech act recognition begins early in the turn when the utterance has only been partially processed, at least when participants are asked to categorize the action of the utterance. This was reflected by frontal positivities from 200 ms after utterance onset in Pre-offers and from 400 ms in Declinations, relative to the control condition (Answers). At the utterance-final word no ERP effects occurred in Declinations, while Pre-offers elicited a late posterior negativity relative to the other actions. The differences between Pre-offers and Declinations, both of which are relatively indirect speech acts, demonstrate that speech act comprehension is not just modulated by indirectness as traditionally construed, but also by the type of action being performed and the relationships between actions in conversation. Below we describe the results in more detail and discuss their implications.

### The time-course of speech act recognition

Conversation is characterized by tight time constraints, allowing listeners limited time between turns (on average only 200 ms) to recognize the speech act and plan an appropriate response [3]. We hypothesized that speech acts are recognized early on in utterances, enabling quick turn transitions. More specifically, we predicted that the critical conditions—Declinations and Pre-offers—would elicit ERP effects relative to the control (Answers) in an early utterance time-window corresponding to the first word and the verb (e.g., *I have*), and not at the utterance-final word (*credit-card*). In line with the first aspect of this prediction, both Declinations and Pre-offers elicited ERP effects in the early utterance time-window. In particular, Pre-offers elicited a frontal positivity relative to Answers in the right hemisphere from 200 to 600 ms after utterance onset. A positivity was also observed in Declinations, relative to Answers, at frontal sites in the right hemisphere from 400 to 500 ms after utterance onset, extending to anterior bilateral and medial regions from 500 to 600 ms.

The earlier onset in Pre-offers is consistent with the results from a self-paced reading study on the same speech acts [35], in which reading times for the first word were longest in Pre-offers. Future studies are needed to assess the functional significance of this early difference between Declinations and Pre-offers. Studies in the visual modality have reported speech act related ERP effects around 100 ms [28,29]. The slightly later onset in the present experiment may be due to noisier language input (spoken vs. visual in previous studies [28,29]) and greater variability in the target utterances (multi-word sentences vs. one word). It is also possible that the effects observed in the early utterance time-window are early responses to the verb, and not the first word. The dialogues involve connected speech with subjects and verbs of relatively short duration; the duration of the first word in the critical utterances was only 141 ms on average (in 89% of cases it was the short pronoun “I” *ik*; see Fig. 1). Importantly, however, the early frontal positivities were absent when time-locked to the final word, indicating that the beginning of the utterance, that is the first word and/or the verb, is critical for this aspect of speech act recognition. These early effects of action type show that, at least when an action categorization task is used, listeners do not wait for the full propositional meaning of the sentence, but proceed immediately with speech act recognition based on the partial information available. This is striking given that the critical utterances do not contain any clues regarding what speech act is being performed.

When is speech act recognition achieved, i.e., when has the speech act been successfully recognized? While the ERP method cannot pinpoint the exact moment in time when recognition occurs, the results for the final word are informative about whether additional processing at the end of the utterance is required. In contrast to our prediction that no ERP effects would occur at the utterance-final word (*credit-card*), a late posterior negativity was observed in Pre-offers from 600 to 1000 ms after final word onset relative to Declinations and from 900 to 1000 ms relative to Answers. This late effect suggests that under certain circumstances listeners do make use of the entire utterance to understand the action. While the final-word effect in Pre-offers is unexpected on an early speech act recognition account, it fits well with our prediction that Pre-offers would elicit additional ERP effects relative to Declinations due to having a more complex action sequence structure. In particular, Pre-offers are less predictable, as the context turn is less constraining (the two turns do not form an adjacency pair). Second, Pre-offers are more predictive, “projecting” further talk in a new action sequence [36]; understanding a Pre-offer involves knowing that a direct offer would follow if encouraged by the recipient. Pre-offers may thus require additional processing based on the complete utterance because of one or both of these characteristics (for a further discussion of the ERP effects, see below).

The prediction that no ERP effects would be observed at the utterance-final word (*credit-card*) was confirmed for Declinations. As discussed in the introduction, the sequential context is highly constraining in Declinations due to adjacency pair structure; given a proposal (*I can lend you money*), there is a normative expectation for either an acceptance or a declination. The first word of the response (*I*…) helps to narrow down the identity of the unfolding speech act by eliminating obvious acceptances of the form *Oh great*, *Phew*! *Thank you* etc. The verb (*I have*…) makes clear that a proto-typical acceptance is not underway, strengthening the likelihood that a declination is involved. By the time the final word is reached, the semantic processing only adds to the propositional meaning, which is the same in all conditions, and hence no ERP differences occur at the final word. We take this to show that in highly constraining contexts—in the present study following the first turn in an adjacency pair—listeners can sidestep the full propositional content of utterances and recognize the speech act before hearing the final word. On the assumption that the action categorization task captures the response demands in everyday conversation, the present results support the early speech act recognition account.

The ERP pattern for Declinations is consistent with the idea that the brain is proactive, constantly generating expectations about upcoming input [50–53]. It has been argued that “we do not interpret our world merely by analysing incoming information, but rather we try to understand it using a proactive link of incoming features to existing, familiar information (e.g., objects, people, situations)” [50]. Past experience with speech act sequences during a lifetime of conversation (stored as scripts in memory [54]), coupled with minimal information about the utterance, may enable listeners to predict the action in advance of its completion. The implication of the present results is that prediction in language comprehension may not be confined to the level of individual lexical items or their syntactic, semantic or conceptual features [51,55–57], but takes place at the level of speech acts as well. Early action recognition through such predictions could be the key to efficient turn-taking, allowing listeners to plan their reply early and respond within the typical 200 ms window between turns in conversation.

Overall, the current findings on the time-course of speech act recognition contribute to a growing body of ERP research (discussed in the introduction) showing that both early and late processes are involved in pragmatic language comprehension. The early effects of action are in line with the view that “different information types (lexical, syntactic, phonological, pragmatic) are processed in parallel and influence the interpretation process incrementally, that is as soon as the relevant pieces of information are available,” [58]. The results extend reports of early pragmatic effects (from 150–200 ms after word onset in discourse studies) [22,23] to dialogue contexts. Fast and incremental processing of speech acts challenges classic theories of pragmatics [19,59] which assume that language understanding proceeds by first extracting the propositional or semantic content of the complete utterance and then comparing that to the context, in order to generate additional pragmatic inferences. However, the results for the utterance-final word—in particular, the late negativity in Pre-offers—highlights that pragmatic language comprehension sometimes involves late inferential processes, as research on irony [24,25] has also shown.

To summarize, we can draw two main conclusions regarding the time-course of speech act recognition. First, even in utterances that are underspecified for the action, speech act recognition begins early in the turn—before the final, critical word has been heard. Second, the time-course of speech act recognition is influenced by how the utterance connects to the larger action sequence. In highly constraining contexts, when the sequence is coming to a close, additional processing at the utterance-final word is not required. This finding is in line with the early speech act recognition account discussed in the introduction. However, in less constraining contexts, when a new sequence is initiated, recognition of the speech act involves analysis of the complete utterance, as reflected by a late posterior negativity at the final word.

### What do the ERP effects reflect?

There are several parallels between our ERP findings and prior research on speech acts and pragmatic language comprehension more generally which we will briefly discuss. The earliest effect of action was observed at frontal sites in the right hemisphere during the early utterance time-window (e.g., *I have*…), from 200 ms after first word onset in Pre-offers and from 400 ms in Declinations. The timing and anterior scalp distribution of this early positivity differentiates it from other positivities reported in the language domain [60–62]. However, the right hemisphere preponderance of this early frontal positivity is consistent with several studies implicating the right hemisphere in pragmatic language comprehension. A visual half-field lexical-decision study found that the right hemisphere plays a critical role in speech act processing in isolated statements such as *Don’t forget to go to the dentist* [63]. Similarly, damage to the right hemisphere causes difficulty with understanding indirect speech acts [64]. EEG and fMRI studies in healthy participants have likewise highlighted the role of the right hemisphere in pragmatic language comprehension [65] and discourse coherence [66]. The right hemisphere advantage in pragmatics has been associated with factors such as coarse semantic coding [67], processing of relatively unpredictable semantic relationships [68], or forming an integrated representation of ongoing discourse [66]. In the MEG study on speech act comprehension previously discussed [29], several activations were found in the right hemisphere. In particular, an effect was observed between 200 and 300 ms in the right inferior frontal gyrus, suggesting that this area plays a role in “binding information about action and context” [29]. At face value, the right hemisphere distribution of the frontal positivity observed in the present study supports the idea that this early effect reflects linguistic and/or discourse-level processes in the right hemisphere that are needed to understand the speech act of the utterance in the given context.

The frontal positivity in Declinations extended to the anterior medial and left hemisphere sites during the early utterance time-window, from 500 to 600 ms after first word onset relative to Answers. This effect is especially prominent at the midline and in the left hemisphere (see waveforms in Fig. 3) and will therefore be referred to as frontal left/midline positivity. While the frontal left/midline positivity in Declinations was not significant relative to Pre-offers (*p* = .06), the waveforms in Fig. 3 suggest that it is absent in Pre-offers. This frontal effect bears some resemblance to a frontal positivity reported in a visual ERP study on indirect requests, which was interpreted as reflecting ease of processing in the indirect condition [30]. However, that interpretation does not fit the present results, as it counter-intuitively implies that Declinations are the easiest out of the three speech acts to process (whereas we expect Answers to be so). What is it about Declinations that triggers this positivity? In contrast to Pre-offers, the context turn in the Declination dialogues builds up strict expectations about the upcoming action (an acceptance or declining of an offer). In contrast to Answers, the Declination target utterances are relatively indirect (providing just a reason for not needing the offer) and therefore more taxing. We propose that this combination of predictability and indirectness triggers the frontal left/midline positivity: listeners quickly “tune in” to the early signals of the action (expecting either declination or acceptance) and yet have to engage in additional processing because of the indirect response. The nature of this additional processing is unclear. Frontal positivities with a left hemisphere and midline distribution have been linked to discourse structure reanalysis [69,70] and update of working memory [71]. The similarity of these positivities to the effect reported here is not obvious, as they are elicited in very different conditions (when a single word is unexpected or pragmatically anomalous). However, an account in terms of discourse structure revision or working memory is compatible with the notion that the beginning of the utterance in Declinations is demanding due to a combination of indirectness and predictability. In particular, the non-canonical beginning of the Declinations (e.g., *I have*…) could trigger revision of the incoming turn in order to make sense of the sentence as a Declination.

An alternative account is that the frontal left/midline positivity reflects socio-emotional factors. As reported in the Introduction, fMRI and MEG studies on indirect speech act comprehension report activations in frontal regions involved in mentalizing, including the medial frontal cortex [20,21,29]. Frontal slow waves similar to the frontal left/midline positivity in Declinations have been reported in studies on theory of mind using narratives or story cartoons [72,73]. Moreover, in supplementary analyses of the present data (presented in supporting information for reasons of space; see S2 File), a correlation was obtained between the frontal left/midline positivity in Declinations and the Empathy Quotient [41], which measures both theory of mind (cognitive empathy) and affective empathy. Although speculative, the frontal left/midline positivity in Declinations may reflect theory of mind processing. Note that frontal theory of mind regions are also activated when participants think about the future [50,74], suggesting a link to prediction: these areas “may be concerned with anticipating what a person is going to think and feel and thereby predict what they are going to do” [74]. The combination of predictability and indirectness in Declinations could trigger theory of mind processing at the beginning of the target utterance, resulting in the frontal left/midline positivity. However, further research is clearly required to investigate the two possible accounts for this ERP effect discussed above.

In addition to the frontal left/midline and right hemisphere positivities observed in the early utterance time-window, a late posterior negativity occurred at the final word (*credit-card*) in Pre-offers. This effect was present in Pre-offers from 600 to 1000 ms after final word onset relative to Declinations, and from 900 to 1000 ms relative to Answers. We interpret this ERP effect as a negativity to Pre-offers rather than as a positivity (P600) associated with Answers and Declinations, since it is not plausible that Answers, the control condition, should require more reanalysis, revision, or pragmatic inferencing—processes traditionally associated with late positivities.

One possibility is that the posterior negativity to the final word in Pre-offers reflects uncertainty associated with the subsequent comprehension task, as the behavioural results indicated that accuracy was descriptively lowest for Pre-offers. The difference in accuracy was due to lower accuracy in stimulus Set 2. The target utterances in Set 1 all start with *I have*…, which is a common way to begin an offer, as attested by studies of English and Finnish [75]. Set 2, on the other hand, is more variable and includes formats such as *I am going*… or *My brother is*…, which may be less common in offering actions and therefore categorized less accurately. If uncertainty in categorization drives the posterior negativity, then the effect should be more pronounced in target sentences in Set 2, for which the task accuracy was lower. However, no differences between Sets were observed in the ERPs, speaking against an uncertainty account for the late ERP effect.

Sustained negativities to sentence-final words have been interpreted as reflecting difficulty in semantic analysis at the message level [76], but this “semantic” sentence negativity peaks earlier than in the present experiment (around 400 ms). Sentence-final negativities have also been linked to working memory operations at clause boundaries (clause-ending negativity, see [77]), in line with studies associating sustained negativities to various linguistic stimuli with increased demands on working memory [78–81]. Negativities associated with working memory have a frontal scalp distribution in most cases and therefore do not match the posterior distribution in the present study (for an exception, see [70]). However, a working memory account for the late negativity in Pre-offers is consistent with the notion that they are more complex than the other two conditions in the study. The context turn in the Pre-offer dialogues is not constraining in terms of what action can follow, and as a consequence the speech act is less predictable. Processing at the final word may be more taxing on working memory in Pre-offers because it requires post-hoc retrieval of the prior turn to figure out what the utterance could mean in that particular context. On this account, a late negativity is not elicited to Declinations, because the prior turn (an offer) has already built up a strong expectation for either an acceptance or a declination, and listeners do not need to compare the utterance to the prior turn in working memory. Whether this working memory account is correct is a topic for further investigation.

### Remaining questions

Some questions relating to conceptual and methodological issues remain. The first concerns the task demands. Overhearing a dialogue is different from taking an active part in a conversation, when participants can be held accountable for both the content and timing of their response. Given that the crucial task in conversation involves recognizing the action in the incoming turn and planning a relevant response, we used an action categorization task to ensure that participants paid as much attention to the actions as in natural dialogue. To some degree this task mirrors the situation in everyday conversation, in which listeners have to make an implicit categorization of each utterance in order to prepare a fitted response. However, participants were given labels of the three speech acts in advance and may have used this top-down information to predict the actions based on the contexts. This raises the question whether, and if so to what extent, task-related processes influenced the ERP effects reported in this article. We are currently investigating this in an ERP study. In this follow-up experiment we use a true-false comprehension task that probes understanding at the message-level without providing information about the speech acts in advance. Important for the present purposes, the main ERP effects reported in the current study are replicated under these different task circumstances. On the assumption that using a comprehension task of some kind better captures the response demands in conversation, the generalization of the current results to a different task environment supports the view that the ERP effects are not induced by the action categorization but rather reflect a more natural aspect of speech act comprehension.

Another issue concerns the role of prosody in speech act comprehension. The target utterances were recorded out of their dialogue contexts (from a list) and used in all three conditions to prevent the prosody from biasing any of the three speech acts. One could argue that this neutral prosody, which included falling intonation, may be better suited for some speech acts than others. For instance, in a test recording during development of the materials, the native speakers sometimes used a fall-rise contour [82] in the Pre-offers. It is therefore possible that a rising intonation is more suitable for the Pre-offers. To what extent the comprehension of the speech acts in the present study was influenced by prosody is not clear. Research on irony has not found an effect of prosody, suggesting that prosody does not necessarily provide crucial cues for utterance interpretation [24]. Whether this holds for speech act comprehension more generally is an empirical question.

## Conclusions

Three main conclusions can be drawn from the present ERP results. First, the early frontal positivities to the target utterance onset show that—at least when participants are asked to categorize the action—speech act recognition begins very early in the incoming turn, starting already from 200 milliseconds after first word onset when the utterance has only been partially processed. This is the case even though the utterance is relatively indirect and contains no morphosyntactic speech act clues. Second, the ERP findings for the utterance-final word reveal that the time-course of speech act recognition is influenced by how the utterance connects to the larger action sequence. In highly constraining (adjacency pair) contexts, when the sequence is coming to a close, no late ERP effect to the final word occurs. This suggests that listeners can recognize the speech act before the final word through predictions at the speech act level. Early speech act recognition may well be the key to efficient conversation, allowing listeners to plan their reply early and respond within the 200 ms time frame characteristic for turn-taking. In some cases, however, additional processing based on the complete utterance is required. This is reflected by a posterior negativity at the utterance-final word when the speech act is in a less constraining context and a new action sequence is initiated. Taken together, the findings show that the time-course of speech act recognition is influenced by the type of action being performed and the sequential context. The present data demonstrate that the speech act dimension is an important aspect of context that should be taken into account in future studies on sentence comprehension in its natural habitat, conversation.

## Supporting Information

S1 File Model comparison for behavioural data analysis. (DOCX)

S2 File Empathy Quotient correlation analysis. (DOCX)

S1 Table Initial omnibus analyses for the early utterance time-window. (DOCX)

S2 Table Initial omnibus analyses for the late utterance time-window. (DOCX)
