# Supplementary material for: Conversation Electrified: ERP Correlates of Speech Act Recognition in Underspecified Utterances
Source: PLoS One. 2015 Mar 20;10(3):e0120068. doi: 10.1371/journal.pone.0120068 (PMC4368040; doi:10.1371/journal.pone.0120068)
Supplement: S1 File — (DOCX) [file pone.0120068.s001.docx]

**Supporting Information:**

**Model comparison for behavioural data analysis**

We first compared the fit of the full model with Action, Set and the Action x Set interaction (AIC 1394.5, logLIK -675.26) to a model without the interaction (AIC 1397.2, logLik -678.58); including the interaction significantly improved the fit of the model (loglikelihood difference = 3.32; χ2(2) = 6.64, p < 0.05). A comparison of a model with Action and Set as fixed effects (AIC 1397.2, logLik -678.58) to a model without Action (AIC 1405.9, logLik -684.97) was significant (loglikelihood difference = 6.39; χ2(2) = 12.76, p < 0.01), suggesting that the best fitting model includes Action as a fixed effect. A comparison of a model with Action and Set as fixed factors (AIC 1397.2, logLik -678.58) to a model with only Action (AIC 1396.5, logLik -679.27) revealed that adding a fixed effect of Set did not significantly improve the model (loglikelihood difference = 0.69; χ2(1) = 1.38, p = 0.24).
