# Supplementary material for: Conversation Electrified: ERP Correlates of Speech Act Recognition in Underspecified Utterances
Source: PLoS One. 2015 Mar 20;10(3):e0120068. doi: 10.1371/journal.pone.0120068 (PMC4368040; doi:10.1371/journal.pone.0120068)
Supplement: S2 File — (DOCX) [file pone.0120068.s002.docx]

**Supporting Information:**

**Empathy Quotient Correlation Analysis**

**Introduction**

Prior fMRI and MEG studies have implicated theory of mind (or mentalizing) and affective empathy in indirect speech act comprehension. For instance, a recent fMRI study found that indirect replies in spoken dialogues (e.g., Did you like my presentation? – It's hard to give a good presentation) activate not only typical language regions but also areas for mentalizing and affective empathy [1]. Similarly, an fMRI study on indirect requests (e.g., It is very hot here presented with a picture of a closed window) reported activations in theory of mind regions, in addition to activations in areas associated with action planning and motor control [2]. Theory of mind activations were also reported in an MEG study on speech act processing, in which written words performed either a requesting or a naming speech act depending on a prior video-taped context sentence (e.g., What are these called?/What can I get you? – Plant) [3]. Based on this research, and the assumption that comprehending the function of a response involves getting a grasp of the intentions behind it, we hypothesized that there might be individual differences in the timing or nature of speech act recognition due to possible variation in perspective-taking and social cognition more generally. Such variation can be assessed with the Empathy Quotient (EQ) [4,5]. The EQ measures both theory of mind (cognitive empathy; the ability to infer the mental state of another person) and affective empathy (the ability to respond emotionally to another person’s emotion) [4]. Theory of mind and affective empathy, as measured with the EQ, have previously been found to influence pragmatic language comprehension in ERP research [6].

Thus to investigate whether theory of mind or affective empathy plays a role in speech act recognition we planned to compute correlations between scores on the Empathy Quotient and the ERP data. More specifically, the goal of the correlation analysis was twofold: to examine whether the observed ERP effects are modulated by empathy, and to check whether individual differences mask other ERP effects that might be present (such as an N400).

**Empathy Quotient scores and correlation analysis**

All participants filled out the Empathy Quotient questionnaire (EQ) after the EEG recording. EQ scores ranged from 20 to 62 (M=40.79, SD=8.46) (highest possible score 80, higher scores indicate more empathy skills). The correlation analysis was performed on mean amplitudes in the early utterance time-window from 100 to 600 ms after first word onset, and in the late utterance time-window from 100 to 600 ms and 600 to 1000 ms after final word onset. The analysis for the early window included the right anterior region and the anterior medial region (to capture modulation of frontal ERP effects), as well as the posterior medial region (to check for effects at posterior sites). The analysis for the final word included the same frontal regions and a posterior region in the left hemisphere (to capture the late negativity). We first averaged the mean amplitude over all sites in each region for each condition. ERP difference scores were then computed for Declinations and Pre-offers by subtracting the mean amplitude for Answers (Declinations - Answers and Pre-offers - Answers, respectively). Two-tailed Pearson’s correlations were performed using Bonferroni-adjusted alpha levels, p = .017 for the early utterance time-window and p = .008 for the two time-windows at the final word.

EQ scores were positively correlated with the Declination difference score at the anterior medial region in the early utterance time-window, i.e., from 100 to 600 ms after first word onset (r(40) = .43, p = .005); the more empathy participants have, the bigger the amplitude difference between Answers and Declinations. A scatter plot showing the correlation is provided in Figure 1. Participants were split into two groups based on EQ scores, resulting in a Low EQ group (20 participants, EQ 20 to 40) and a High EQ group (22 participants, EQ 41 to 62). Visual inspection of both groups’ mean waveforms for Answers and Declinations at the first word (see Figure 1) suggests that Declinations separate earlier from Answers in participants with high empathy than in low empathizers. Separate analyses (ANOVAs) for each group in the early utterance time-window revealed that the frontal positivity at the medial region is present from 400 to 600 ms in the High EQ group, indicated by a main effect of Action at the frontal medial region (Fs ≥ 7.06, ps < .05), while no differences were observed in the Low EQ group.

The ERP difference scores in Pre-offers were not significantly correlated with EQ at the first word (ps > .10). At the final word there were no significant correlations between EQ and ERP difference scores using the corrected alpha level (ps > .016; the corrected alpha level was .008 in the late time-windows).

**Discussion**

A positive correlation was observed between EQ and the Declination difference score at the frontal medial region from 100 to 600 ms after first word onset, indicating that the more empathy participants have, the larger the mean amplitude difference between Answers and Declinations. The follow-up analyses indicated that the frontal medial positivity (referred to as frontal left/midline positivity in the main article) is present from 400 to 600 ms after first word onset in high empathizers only. The correlation suggests that the frontal medial positivity is related to theory of mind and/or affective empathy. Unfortunately it is difficult to disentangle the cognitive and emotional components of empathy as they tend to co-occur [4]. Prior fMRI research indicates that comprehension of indirect speech acts activates both mentalizing and affective empathy areas [1,2], as previously discussed.

In the main article we speculate that the correlation with the Empathy Quotient in Declinations reflects individual differences in theory of mind processing. Declinations may be more taxing on theory of mind than Pre-offers and Answers due to a combination of predictability and indirectness. In contrast to Pre-offers, the context turn in the Declination dialogues builds up strict expectations about the upcoming action (an acceptance or declining of an offer). In contrast to Answers, the Declination target utterances are relatively indirect (providing just a reason for not needing the offer) and therefore more difficult. These factors may trigger theory of mind processing in Declinations, reflected by the frontal medial positivity; listeners quickly “tune in” to the early signals of the action (expecting either declination or acceptance) and yet have to engage in additional processing because of the indirect response. On this account the time-course of speech act recognition is influenced by theory of mind abilities. However, further research is clearly required to investigate the influence of cognitive and emotional empathy on the time-course of speech act recognition.

**Figure 1.** **Empathy Correlation.** A) Scatter plot showing correlation between EQ and Declinations difference score in the early utterance time-window at the frontal medial region. B) ERPs at Fz time-locked to the onset of the first word; for all participants, high empathy participants, and low empathy participants.


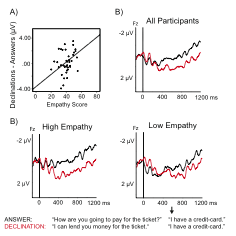


**References**

1. Basnakova J, Weber K, Petersson KM, Van Berkum JJA, Hagoort P (2014) Beyond the language given: The neural correlates of inferring speaker meaning. Cerebral Cortex 24: 2572–2578.

2. Van Ackeren MJ, Casasanto D, Bekkering H, Hagoort P, Rüschemeyer SA (2012) Pragmatics in action: Indirect requests engage theory of mind areas and the cortical motor network. Journal of Cognitive Neuroscience 24: 2237–2247.

3. Egorova N, Pulvermüller F, Shtyrov Y (2013) Neural dynamics of speech act comprehension: An MEG study of naming and requesting. Brain Topography 27: 375–392.

4. Baron-Cohen S, Wheelwright S (2004) The empathy quotient: An investigation of adults with Asperger syndrome or high functioning autism, and normal sex differences. Journal of Autism and Developmental Disorders 34: 163–175.

5. Lawrence EJ, Shaw P, Baker D, Baron-Cohen S, David AS (2004) Measuring empathy: Reliability and validity of the Empathy Quotient. Psychological medicine 34: 911–920.

6. Van den Brink D, Van Berkum JJA, Bastiaansen M, Tesink CM, Kos M, et al. (2012) Empathy matters: ERP evidence for inter-individual differences in social language processing. Soc Cogn Affect Neurosci 7: 173–183.
