# Supplementary material for: Conversation Electrified: ERP Correlates of Speech Act Recognition in Underspecified Utterances
Source: PLoS One. 2015 Mar 20;10(3):e0120068. doi: 10.1371/journal.pone.0120068 (PMC4368040; doi:10.1371/journal.pone.0120068)
Supplement: S1 Table — (DOCX) [file pone.0120068.s003.docx]

**Supporting Information**

**Table S1**

**Initial omnibus analyses for the early utterance time-window**

| Analysis | Source | DF | 100-200 | 200-300 | 300-400 | 400-500 | 500-600 |
| --- | --- | --- | --- | --- | --- | --- | --- |
| Lat | Action×Hem×Site | 24, 984 | 2.77** | 3.34** | 2.33** | 2.48** | 2.83** |
| Mid | Action×Site | 6, 246 |  |  |  | 3.45* | 4.63** |

*Lat = lateral sites, Mid = midline sites, Hem = hemisphere. *p < .05, **p<.01.*
