# Supplementary material for: Conversation Electrified: ERP Correlates of Speech Act Recognition in Underspecified Utterances
Source: PLoS One. 2015 Mar 20;10(3):e0120068. doi: 10.1371/journal.pone.0120068 (PMC4368040; doi:10.1371/journal.pone.0120068)
Supplement: S2 Table — (DOCX) [file pone.0120068.s004.docx]

**Supporting Information**

Table S2

Initial omnibus analyses for the late utterance time-window, for those epochs that showed significant effects.

| Analysis | Source | DF | 100-200 | 600-700 | 700-800 | 800-900 | 900-1000 |
| --- | --- | --- | --- | --- | --- | --- | --- |
| Lat | Action×Site | 24, 984 |  | 2.43* | 2.68* | 2.51* | 2.74* |
|  | Action×Hem | 2, 82 | 3.43* |  |  |  |  |

Note: *Lat = lateral sites, Hem = hemisphere. *p < .05, **p<.01.*
